# Corrigendum to “Rehabilitation Training and Resveratrol Improve the Recovery of Neurological and Motor Function in Rats after Cerebral Ischemic Injury through the Sirt1 Signaling Pathway”

**DOI:** 10.1155/2017/1532462

**Published:** 2017-07-02

**Authors:** Na Shi, Chongtian Zhu, Liying Li

**Affiliations:** ^1^Rehabilitation Department, Linyi People's Hospital, Linyi, China; ^2^Department of Rehabilitation, Shandong Medical College, Linyi, China

In the article titled “Rehabilitation Training and Resveratrol Improve the Recovery of Neurological and Motor Function in Rats after Cerebral Ischemic Injury through the Sirt1 Signaling Pathway” [1], affiliation number two was given incorrectly. The corrected affiliation is shown above.
